# Special Issue “Cellular Redox Mechanisms in Inflammation and Programmed Cell Death”

**DOI:** 10.3390/ijms27010274

**Published:** 2025-12-26

**Authors:** Irina I. Vlasova

**Affiliations:** Institute for Regenerative Medicine, Sechenov First Moscow State Medical University (Sechenov University), 119991 Moscow, Russia; iivlasova08@gmail.com or vlasova_i_i@staff.sechenov.ru

## 1. Introduction

**Figure 1:**
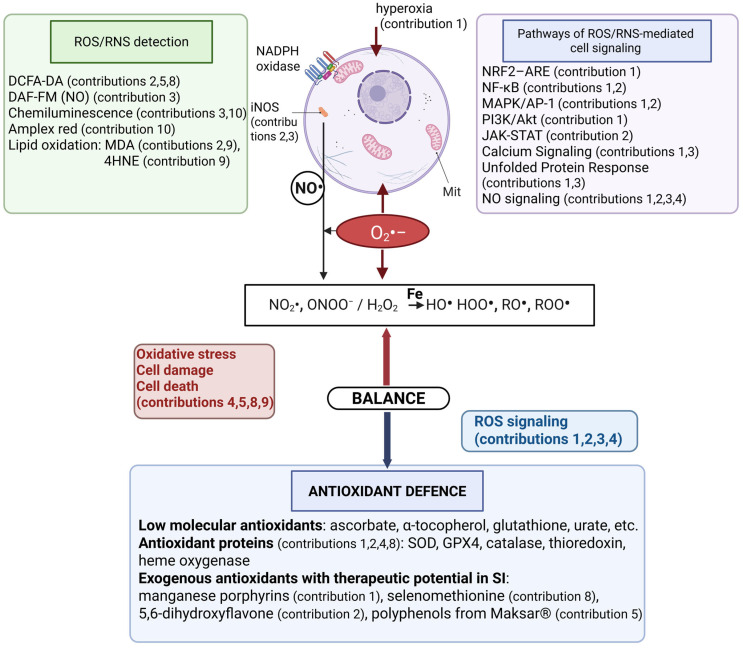
The balance between ROS/RNS and antioxidant system determines the involvement of reactive species participation in cell signaling or cell damage. The top part of the scheme presented ROS and RNS, the sources of their formation in cells, methods of RONS detection, and signaling pathways. The lower part of the scheme presented antioxidant systems—exogenous low molecular compounds, antioxidant enzymes, and compounds proposed in SI papers capable of buffering redox imbalance. References from the SI are provided for each topic. Abbreviations are listed at the end of text. Mit—mitochondria.

Redox reactions play a central role in the regulation of physiological processes in cells and tissues. Reactive oxygen species (ROS) and reactive nitrogen species (RNS) are finely integrated in cellular signaling pathways [1]. They participate in gene regulation, cellular communication and host defense, orchestrating the balance between cellular homeostasis and stress response [2,3]. The main source of ROS is plasma membrane NADPH oxidase [4]. The enzyme catalyzes the formation of superoxide anion (O_2_•−), which is the primary ROS formed from molecular oxygen. It is unstable and dismutates spontaneously, or is converted by superoxide dismutase (SOD) into hydrogen peroxide [5]. H_2_O_2_ is a stable compound capable of migrating relatively long distances until it encounters iron in low-molecular iron complexes or in the active centers of some enzymes initiating the formation of other ROS (Figure 1) [6]. The primary RNS is nitric oxide (NO•, NO), which is synthetized in cells by nitric oxide synthases (NOSs) [7]. NO• is a relatively stable compound with an array of health benefits: control of cardiovascular system through modulation of vasodilation, regulation of immune response, neurotransmission, and inhibition of ferroptosis [8,9]. When interacting with superoxide anion, NO• is converted into highly reactive peroxynitrite, switching the biochemistry of NO• toward nitro-oxidative processes [10].

The innate immune response is initiated by “alarm signals” from the damaged tissues and pathogens. The main participants in the immediate and non-specific innate immunity are neutrophils, monocytes, and macrophages [11]. They are professional phagocytes, and their main functions are ROS production and phagocytosis of pathogens and dead cells. Macrophages participate in and orchestrate all stages of inflammation. The traditional M1/M2 paradigm distinguishes two polar macrophage subtypes based on their phenotypic characteristics, cytokine secretion, and transcriptional profiles: classically activated M1 phenotype and alternatively activated M2 macrophages [12]. M1 macrophages produce ROS and pro-inflammatory cytokines to promote inflammation [13]. M2 macrophages exhibit anti-inflammatory and immunoregulatory activity mediating resolution of inflammation and tissue regeneration [14].

Along with low molecular antioxidants, antioxidant enzymes play a crucial role in maintaining cellular redox homeostasis. SOD, catalase, glutathione peroxidases (GPX), thioredoxin, and heme oxygenase control the redox status of the cells. The search for bioactive molecules capable of buffering redox imbalance is a critical task [15]. Flavonoids are an important family of polyphenolic compounds classified as secondary plant metabolites and widely present in fruits and vegetables. Numerous studies have demonstrated their protective role in pathologies caused by oxidative stress [16]. Exogenous antioxidants with therapeutic potential, which introduces the concept of hormesis applied to flavonoids, also called "hormetic nutrients", induce health benefits by activating the Nrf2 pathway and related antioxidant enzymes for protection during oxidative damage, apoptosis/ferroptosis, and chronic inflammatory disorders [17,18]. The optimal dose, which is the minimum dose capable of producing beneficial effects, is crucial to achieve protection and prevent harmful effects and must be carefully identifying.

When ROS/RNS production in cell and tissues exceeds antioxidant capacity, reactive species initiate oxidative damage through modification of lipids, proteins, and nucleic acids, triggering pathology of tissues and programs of cell death [19]. Apoptosis is the most common form of cell death for damaged or aged cells. The primary mechanism for apoptosis is caspase activation, and its hallmark is cytochrome *c* (cyt *c*)-induced oxidation of mitochondrial cardiolipin [20]. Cyt *c* and other mitochondrial pro-apoptotic factors released into cytosol, where cyt *c* can bind to and oxidize an anionic phosphatidylserine (PS) changing the cell membrane properties. PS and oxidized PS exposed to cell surface serve as eat me signals in efferocytosis [21]. The term “ferroptosis” comes from the dependence of this type of cell death on intracellular iron. The program’s key regulatory mechanisms involve the Fe-dependent generation of phospholipid hydroperoxides and their reduction to alcohols by GPX4 via the oxidation of glutathione [21,22,23].

The Special Issue (SI) contains two reviews and eight original research. The goal of the Editorial is to summarize and classify the contents of all the studies in order to guide the readers through the SI book (Figure 1, Table 1).

## 2. An Overview of Published Articles

The articles presented in the SI highlight the pathways of cellular stress response to bacterial infections, toxic compounds, metabolic imbalance, or cell cooling. The articles do not merely highlight the mechanisms of redox-dependent damages but show how targeted antioxidant strategies can restore cellular homeostasis, considering the redox distress as both a driver of pathology and a target for therapeutic intervention.

The review by Silva et al. evaluates the role of reactive species in cell signaling and pathologies, focusing on neuropathic pain (contribution 1). The first part of the review can serve as an Introduction to the SI. This article is suitable for introducing redox reactive species, the redox interactions and regulations in cells. For each ROS and RNS type (RONS), the chemical processes and reactions are described. The authors considered the sources of ROS in cells: plasma membrane NADPH oxidase and the mitochondrial transport chain, as well as, to a lesser extent, the endoplasmic reticulum, peroxisomes, and cytosol. The review summarizes mechanisms of cell signaling, and discusses the mechanisms of antioxidant enzyme activity.

### 2.1. Pathways of ROS/RNS-Mediated Cell Signaling

RONS act as mediators of cellular signaling pathways that are involved in the modulation of normal physiological functions and the initiation of the response to cellular injury.

The mechanisms of activation of the main ROS/RNS-mediated cellular signaling pathways and their role in neuropathic pain are discussed in the review (contribution 1) (Section 2.2.1, Section 2.4, and Section 2.5). The authors provide a detailed description and diagrams of nuclear factor erythroid 2-related factor 2/antioxidant response elements (NRF2–ARE), nuclear factor kappa-B (NF-κB), mitogen-activated protein (MAPK/AP-1) and phosphoinositide 3-Kinase (PI3K)/Akt) signaling pathways, calcium signaling, and unfolded protein response. The mediating factors are translocated to the nucleus where they activate gene transcription and signal transduction.

Activation of the cell signaling pathways was studied in RAW 264.7 macrophages treated with lipopolysaccharides (LPS) (contribution 2). The interaction of LPS with the cell surface toll-like receptor 4 (TLR4) triggers the activation of signaling cascades, including the NF-κB, MAPK pathways, and the Janus kinase (JAK)-signal transducer and activator of the transcription (STAT) pathway. Protein phosphorylation is an important mechanism underlying the activation of all of these pathways and the transmission of cellular signals. Blocking TLR4 activation with 5,6-dihydroxyflavone suppresses cell activation by inhibiting the phosphorylation of c-JNK and p38 in the MAPK pathway, JAK2 and STAT3 in the JAK-STAT pathway, and p65 from the NF-κB pathway (Section 2.4 and Section 2.5). Importantly, MAPK, JAK-STAT, and NF-κB are non-overlapping pathways, but they act in concert to regulate the macrophage response to LPS.

Endoplasmic reticulum (ER) stress accompanied by the accumulation of unfold-ed/misfolded proteins in the ER lumen induces the unfolded protein response (UPR), which aims to resolve ER stress or initiates cell death if ER stress cannot be resolved [24,25]. However, the biological effects of ER stress are not always mediated by UPR. Induction of ER stress in liver tissue by the antibiotic tunicamycin resulted in vasodilation of blood vessels in hepatic parenchyma in an unconventional way independent of UPR (contribution 3). Vasodilation can be caused by ER stress itself due to disruption of Ca^2+^ homeostasis and triggering of NO-generation mechanisms, which are key regulators of vascular tone. Interestingly, the elevated NO level is provided by its release from intracellular NO store in smooth muscle cells, rather than from endothelial NOS activity (Section 2.2.3).

### 2.2. ROS/RNS-Related Pathological Conditions

Oxidative stress and inflammation are often inseparable partners in response to infections or injury. If left uncontrolled, these processes lead to tissue disfunction and the development of pathologies.

#### 2.2.1. Neuronal Disorders

Nitroxidative stress in neuronal cells involves the complex interactions between RONS, antioxidant systems, and cell signaling that underlie the pathophysiology of neuropathic pain (contribution 1) (Section 2.1, Section 2.4, and Section 2.5). Immune cells of the central nervous system play a crucial role in the development and maintenance of neuropathic pain as nerve injury results in a neuroinflammatory response and activation of glial cells. Microglia, which are resident macrophages in the brain, initiate the development of neuropathic pain by releasing inflammatory mediators that not only transmit signals to other cells but also cause cell damage and neuronal death. Activated glial cells release chemokines, ROS, and neurotransmitters that modulate synaptic transmission, causing pain amplification and promoting the death of dopaminergic neurons.

Glutamate, a vital mitochondrial substrate fueling oxidative phosphorylation, also plays a central role in neuronal signaling [26], but high levels of extracellular glutamate are toxic, causing dysregulation of intracellular redox homeostasis, mitochondrial dysfunction, and cell death (contribution 4) (Section 2.3). Under physiological conditions, glutamate is stored inside cells in what’s known as an easily releasable pool. In pathological conditions such as neuroinflammation, brain trauma, and neurodegenerative diseases, glutamate concentrations in extracellular space increase sharply. Disruption of glutamate metabolism in mitochondria leads to a vicious cycle: disruption of the tricarboxylic acid cycle due to neuroinflammation or elevated cortical NO level increases glutamate concentration, which leads to mitochondria damage due to the impaired oxidative phosphorylation [27].

Treatment of Neuro-2a cells by 6-hydroxydopamine (6-OHDA) and paraquat (PQ) caused an increase in intracellular ROS, a decrease in mitochondrial membrane potential, and apoptotic cell death (contribution 5) (Section 2.2.3, Section 2.3 and Section 2.4). Herpes simplex virus type 1 (HSV-1) infection is a risk factor for neurodegenerative diseases, primarily due to the virus-induced mitochondrial disfunction, accumulation of ROS in neurons, and lipid peroxidation.

#### 2.2.2. Microbiota-Related Metabolites in Inflammation and Pathologies

The human microbiota produces metabolites that can enter the bloodstream and participate in the regulation of immune system, central nervous system, and metabolism.

Cyclooxygenase (COX) is one of the key enzymes in the inflammatory process. It catalyzes the synthesis of prostaglandin H2, a precursor of all prostaglandins and thromboxanes [28,29]. The influence of some selected compounds from microbiota-related metabolites on the activity of COX was tested using pure enzyme and in monocyte lysates (contribution 6). Microbial metabolites lowered the activity of COX mainly due to their effect on the peroxidase activity of the enzyme. The study demonstrates how systemic inflammation may be fine-tuned by small molecules of microbial origin. Under septic conditions, the metabolites may shift COX peroxidase activity, modulating prostaglandin synthesis and the downstream inflammatory cascade.

Risk factors for cardiovascular diseases are associated not only with metabolic syndrome but also with some gut microbiota metabolites, including trimethylamine-N-oxide (TMAO), which stimulates an active search for TMAO inhibitors (contribution 7). Hypoxic conditions significantly and multifacetedly alter the ratio of intestinal microbiome strains and their metabolites in feces and blood. Intermittent normobaric hypoxic–hyperoxic exposures (IHHEs) are successfully used in the treatment of various pathologies. Moderate hypoxic conditions can activate various transcriptional pathways that initiate the synthesis of proteins with antioxidant activity, while episodes of hyperoxia activate ROS-dependent mechanisms stimulating the antioxidant defense systems of cells (Figure 1). The prospective randomized pilot study demonstrated that IHHEs tend to reduce blood TMAO and cause a more significant reduction in cardiometabolic and hepatic markers of metabolic syndrome compared with the placebo group.

#### 2.2.3. Cellular and Tissue Models of Pathologies

Models of 6-OHDA and PQ-induced neurotoxicity in Neuro-2a cells and HSV-1 infection in Vero cells were used (contribution 5) (Section 2.2.1, Section 2.3, and Section 2.4).

Mammary epithelial cells (bMECs) were infected with Nocardia cyriacigeorgica to create a bovine mastitis model (contribution 8) (Section 2.3 and Section 2.4). Mastitis in cattle reduces milk quantity and quality, and it is accompanied by mammary dysfunction. The bacteria-induced oxidative stress and inflammatory response in bMECs accompanied by elevating mRNA production and the expression of the key pro-inflammatory cytokines, such as tumor necrosis factor-α (TNF-α), interleukin (IL)-1, IL-6, and IL-8, decreasing the activity of antioxidants enzymes such as SOD and GPX and generating destructive levels of malondialdehyde (MDA) and ROS. This resulted in mitochondrial damage and apoptosis in cells.

Precision cut tissue slices have emerged as a physiologically relevant ex vivo model for studying human diseases. Precisely cut liver tissue slices preserving both liver structure and cellular functions were used to study the functionality of hepatic vessels and their response to various treatments (contribution 3) (Section 2.1). The effect of antibiotic tunicamycin was similar to that of specific vasodilator acetylcholine: both compounds induced ER stress in all types of liver cells, disrupting Ca^2+^ transport and increasing NO level, and caused vasodilation of liver vessels increasing their volume. Unlike acetylcholine, tunicamycin did not induce NO release in HUVECs cells, where endothelial NOS is the sole source of NO. Possibly, tunicamycin induces NO production in the smooth muscle layer of the vessels.

### 2.3. ROS-Mediated Programmed Forms of Cell Death: Apoptosis and Ferroptosis

Although reactive oxygen species are necessary for physiological processes, their excess can cause cell damage and trigger a program of cell death.

After infection of bMECs with N. cyriacigeorgica, MDA and ROS levels were significantly upregulated, and cell damage and apoptosis increased in a time-dependent manner (contribution 8) (Section 2.2.3 and Section 2.4). Transmission electron microscopy revealed mitochondrial and endoplasmic reticulum swelling, loss of organelles, cell membrane rupture, cristae degeneration, all markers of apoptosis. Bacteria were detected in the nucleus and cytoplasm of the cells. Annexin V-FITC/propidium iodide assay confirmed apoptotic cell death in infected bMECs. Selenomethionine inhibits bacteria-induced apoptosis in bMECs (Section 2.4).

In Neuro-2a cells, a significant increase in the percentages of Annexin V-detected early and late apoptotic cells was observed after treatment with 6-OHDA (contribution 5). Polyphenols extracted from Maackia amurensis heartwood are registered in the Russian Federation as a drug named Maksar^®^. Some of these polyphenols reduced the neurotoxic effect of 6-OHDA treatment (Section 2.2.1, Section 2.2.3, and Section 2.4).

Human Embryonic Kidney (HEK) 293 cells were studied during a cooling–rewarming procedure (contribution 9). University of Wisconsin Cold Storage Solution substantially increased cell survival during hypothermia (4°C) compared to DMEM, but upon rewarming, lipid peroxidation and ATP depletion rapidly occurred, leading to complete cell death. Cell death was not prevented by an apoptosis inhibitor. In contrast, blocking of ferroptosis by the specific ferroptosis inhibitor ferrostatin-1 or maintaining mitochondrial functions by the 6-chromanol SUL150 effectively prevented ferroptosis. The study provides new approaches for organ preservation in clinical transplantation surgery.

Primary pathways of neuron death induced by glutamate include excitotoxicity and ferroptosis (contribution 4) (Section 2.2.1). High levels of extracellular glutamate block the cysteine–glutamate antiporter, leading to the depletion of intracellular cysteine and subsequently glutathione. The depletion of glutathione inactivates GPX-inducing oxidation of lipids and ferroptosis.

### 2.4. Exogenous Antioxidants with Therapeutic Potential

An important theme of the issue is the search for bioactive molecules capable of buffering redox imbalance and control inflammation.

Manganese porphyrins (MnPs) are a class of low molecular weight synthetic metalloproteins containing manganese in the porphyrin ring at different oxidative states (contribution 1) (Section 2.1, Section 2.2.1, and Section 2.5). Initially, MnPs were considered simply as O_2_•− scavengers (as SOD mimics), but new knowledge about their redox chemistry has revealed that MnPs influence signaling pathways and cellular functions such as proliferation, differentiation, and cell death. Unlike iron porphyrins, MnPs have a very limited ability to react with H_2_O_2_ and produce the dangerous hydroxyl radical (HO•) in a Fenton-like reaction.

Selenomethionine (SeMet) is a form of selenium with antioxidant properties. The therapeutic effect of SeMet at different concentrations was studied in a mastitis model using bMECs infected with N. cyriacigeorgica, which is resistant to most antibiotics (contribution 8) (Section 2.2.3 and Section 2.3). Pretreatment of cells with SeMet mitigated bacterial-induced inflammatory response and oxidative damage in bMECs by reducing MDA and ROS concentrations, decreasing the release of pro-inflammatory cytokines, upregulating SOD and GPX activities, and reducing apoptosis.

5,6-dihydroxyflavone (5,6-DHF) is a flavonoid with anti-inflammatory and antioxidant activity. In RAW 264.7 macrophages, 5,6-DHF significantly blocked LPS-induced activation of cell surface receptor TLR4, preventing stimulation of cell activation pathways (contribution 2) (Section 2.1 and Section 2.5). 5,6-DHF reduced cytoplasmic ROS production in a dose-dependent manner, blocked mtROS formation, hindered the expression of iNOS, thereby reducing NO release, and suppressed the expression of pro-inflammatory cytokines IL-1β, IL-6, and TNF-α. Furthermore, 5,6-DHF scavenged ROS and induced heme oxygenase-1 expression. 5,6-DHF had the highest anti-inflammatory and antioxidant properties in RAW 264.7 cells compared to its natural analogues, baicalein and chrysin.

Maksar^®^, a polyphenol-based drug, possesses hepatoprotective, antithrombogenic, antiplatelet, antitumor, and antioxidant properties (contribution 5). Fourteen compounds were isolated from the drug and tested for neuroprotective potential and anti-HSV-1 activity (Section 2.2.1, Section 2.2.3, and Section 2.3). The most active compounds were found. These compounds regulated mitochondrial membrane potential and significantly reduced intracellular ROS level increasing the viability of 6-OHDA-treated Neuro-2a cells. Maksar^®^ is therefore a promising drug for the treatment of herpetic infections and neuronal pathologies.

### 2.5. Macrophages

Macrophages are the main participants and conductors of all stages of inflammation. Under the influence of tissue factors, they can change their phenotype over a wide range between the M1 and M2 states, which represent the polar phenotypes in the continuum of phenotypic variability of macrophages. When characterizing the macrophage phenotype, the main focus is usually on secreted cytokines and expressed membrane proteins: THF-, IL-6, and CD86 are markers of M1 phenotype, while IL-10, IL-1Ra, and CD206, CD163 are markers of M2 phenotypes (contribution 10). Studies of four different macrophage phenotypes revealed their differences not only in surface markers and cytokine profiles but also in their functional activity, namely radical-generating and phagocytic activities, and even in mechanical properties. This extends our appreciation of the M1/M2 dichotomy toward a continuum of functional states with varying combinations of both markers and functional activities.

Morphologically, microglial cells are described as having three phenotypes similar to those in peripheral macrophages: M0 phenotype for the resting state, M1 and M2 phenotypes for activated cells (contribution 1) (Section 2.1, Section 2.2.1, and Section 2.4). The M0 state is characterized by the expression of genes associated with neuronal function and development, whereas the M1 and M2 macrophages are induced by CNS injury and chronic pain, appearing as mixed, time-varying phenotypes that are not in equilibrium on an activation continuum.

Bacterial LPS interacts with TLR4 receptor on the surface of RAW 264.7 cells and triggers inflammatory cascade in murine macrophages through the key signaling pathways (contribution 2) (Section 2.1 and Section 2.4).

## 3. Conclusions

Exploring the articles of the SI provides a panoramic view of redox biology as a unifying concept encompassing seemingly disparate fields: infection biology, immunology, vascular physiology, metabolism, transplantation, and neurology. Each article of the SI addresses a distinct topic of redox biology, but together they reinforce the central principle that redox signals are integral to every stage of inflammation and cell death, from initiation and progression to resolution, which makes them key targets for controlling inflammation, preventing tissue damage, and promoting repair.

## Figures and Tables

**Table 1:** Summary of topics highlighted in the articles of the Special Issue. “Sections” are the Sections in the text where the articles are discussed.

Contribution/ Sections in Chapter 2.	Cells/Model	ROS, RNS Activation	Pathology/ Problem	ROS/RNS Detection	A Form of Cell Death	**Antioxidants/** **Effectors**
(contr. 1) Review Silva et al. Section 2.1, Section 2.2.1, Section 2.4, and Section 2.5	neurons and glial cells (including microglia)	-	neuronal pain	review of all RONS, their chemistry and origins, mechanisms	-	manganese porphyrins
(contr. 2) Cao et al. Section 2.1, Section 2.4 and Section 2.5	RAW 264.7 macrophages	LPS	inflammation	DCFH-DA, mtROS—MitoSOX red; MDA	-	5,6-dihydroxy flavone
(contr. 3) Zavadskis et al. Section 2.1 and Section 2.2.3	precisely cut liver slices HUVECs	NO release: tunicamycin acetylcholine	tunicamycin effects on liver	chemiluminescence DAF-FM		NO-dependent vasodilation
(contr. 4) Review Vaglio-Garro et al. Section 2.2.1 and Section 2.3	neurons, astrocytes	mitochondria disfunctions	brain pathologies		ferroptosis	
(contr. 5) Tarbeeva et al. Section 2.2.1, Section 2.2.3, Section 2.3, and Section 2.4	neuro-2a cells Vero cells	6-OHDA, paraquat; HSV-1 virus	neurotoxicity	DCFH-DA	apoptosis	polyphenols from amurensis heartwood (Maksar^®^)
(contr. 6) Beloborodova et al. Section 2.2.2	COX Lysates of THP- 1, monocytes		sepsis	COX activity, TMPD		microbiota metabolites
(contr. 7) Bestavashvili et al. Section 2.2.2	a prospective randomized study, TMAO is a risk factor of CVDs	hypoxic–hyperoxic exposures	cardiovascular diseases and metabolic syndrome			hyperoxia activates the antioxidant defense systems of cells
(cont. 8) Assabayev et al. Section 2.2.3, Section 2.3 and Section 2.4	mammary epithelial cells (bMECs)	Nocardia cyriacigeorgica	bovine mastitis	DCFH-DA	apoptosis	seleno methionine
(contr. 9) Gartzke Section 2.3	Human Embryonic Kidney (HEK) 293 cells	cell cooling- rewarming	cell preservation	Western blot against 4HNE and MDA measurements	ferroptosis	ferrostatin-1, 6-chromanol SUL150— mitochondrial functions
(contr. 10) Suleimanov Section 2.5	myeloid-derived macrophages	PMA, opsonized zymosan	Inflammation	chemiluminescence Amplex red		

**Abbreviations**: Contr.—contribution; CVDs—Cardiovascular Diseases; 4HNE—4-Hydroxy-Nonenal; DAF-FM—4-Amino-5-Methylamino-2′,7′-Difluorofluorescein Diacetate; DCFH-DA—2,7-Dichlorodihydrofluorescein Diacetate; PMA—Phorbol 12-Myristate 13-Acetate; TMPD—N,N,N′,N′-Tetramethyl-p-Phenylenediamine; TMAO—Trimethylamine-N-Oxide.

## Abbreviations

5,6-DHF—5,6-Dihydroxyflavone; 6-OHDA—6-Hydroxydopamine; AP-1—Activator Protein 1; bMECs—Bovine Mammary Epithelial Cells; CNS—Central Nervous System; Contr—Contribution; COX—Cyclooxygenase; ER—Endoplasmic reticulum; GPX—Glutathione Peroxidases; HSV-1—Herpes Simplex Virus Type 1; IHHEs—Hypoxic–Hyperoxic Exposures; IL—interleukin; JAK-STAT—Janus Kinase-Signal Transducer and Activator of Transcription; LPS—Lipopolysaccharides; MAPK—Mitogen-Activated Protein Kinase; MDA—Malondialdehyde; Mit—mitochondrion, MnPs—Manganese Porphyrins; NO—Nitric Oxide; NO_2_•—Nitrogen Dioxide; NOSs—Nitric Oxide Synthases; NF-κB—Nuclear Factor Kappa-B; O_2_•−—Superoxide Anion; ONOO–—Peroxynitrite; 6-OHDA—6-hydroxydopamine; PI3K—Phosphoinositide 3-Kinase; PQ—Paraquat; RNS—Reactive Nitrogen Species; ROS—Reactive Oxygen Species; RONS—Reactive Oxygen and Nitrogen Species; mtROS—mitochondrial Reactive Oxygen Species; SeMet—Selenomethionine; SI—Special Issue; SOD—Superoxide Dismutase; TLR4—toll-like receptor 4; TNF-α—tumor necrosis factor-α; UPR—Unfolded Protein Response.
